# Predictive value of live birth rate based on different intrauterine adhesion evaluation systems following TCRA

**DOI:** 10.1186/s12958-021-00697-1

**Published:** 2021-01-22

**Authors:** Mingzhu Cao, Yingying Pan, Qingyan Zhang, Danming You, Shuying Feng, Zhi Liu

**Affiliations:** 1 Department of Obstetrics and Gynecology, Center for Reproductive Medicine, Key Laboratory for Major Obstetric Diseases of Guangdong Province, The Third Affiliated Hospital of Guangzhou Medical University, Guangzhou, China; 2 Department of Ultrasound, Nanfang Hospital, Southern Medical University, Guangzhou, 510515 China; 3 The First School of Clinical Medicine, Southern Medical University, Guangzhou, China; 4 Department of Obstetrics and Gynecology, Sun Yat-sen Memorial Hospital, Sun Yat-sen University, Guangzhou, 510120 China

**Keywords:** Intrauterine adhesion, Transcervical resection of adhesion, Live birth, Nasr classification

## Abstract

**Objective:**

The aim of this study was to assess the predictive value of five different intrauterine adhesion (IUA) evaluation systems for live birth rate following transcervical resection of adhesion (TCRA).

**Method:**

This retrospective study included 128 women with IUA who desired for spontaneous conception after TCRA. All the patients were retrospectively scored by the American Fertility Society (AFS) classification, European Society of Gynecological Endoscopy (ESGE) classification, March’s classification (March), Nasr classification (Nasr) and Chinese IUA diagnosis classification criteria (Chinese). The predictive value of these evaluation systems was determined by receiver operating characteristic (ROC) curves and area under a ROC curve (AUC).

**Results:**

The correlation coefficients of AFS, ESGE, March, Nasr and Chinese classification and the live birth rate were 0.313, 0.313, 0.288, 0.380, and 0.336, respectively. Among women with hypomenorrhea and amenorrhea, as well as women with no infertility, the severities determined by all five evaluation systems were correlated with live birth rate (*P* <  0.001). All five scoring systems were efficient to predict live birth rate. Among them, Nasr classification showed the highest AUC (0.748) with the best predictive value. Multivariate logistic regression analyses showed that Nasr classification had the highest OR (OR, 6.523; 95% CI, 2.612, 18.263). And, Nasr’s classification system also showed highest sensitivity (81.8%) and negative predictive value (96.7%) when divide the system into mild IUA vs. moderate and severe IUA.

**Conclusion:**

AFS, ESGE, March, Nasr and Chinese classification were demonstrated to be capable of predicting live birth following TCRA although the predictive capacities might be limited, and Nasr classification showed the highest predictive value of live birth.

**Supplementary Information:**

The online version contains supplementary material available at 10.1186/s12958-021-00697-1.

## Introduction

Intrauterine adhesion (IUA) is also called Asherman’s syndrome (AS), which is defined by the presence of adhesions in the uterine endometrium, and lead to hypomenorrhea, amenorrhea, subfertility, miscarriage and abnormal placentation [1]. The dominant risk factors of IUA is surgical trauma, such as frequent hysteroscopic surgery, curettage of the basalis layer of the endometrium [1], induced abortion, and myomectomy [2]. Once the basalis layer of the endometrium is injured, fibrous tissue forms with a loss of stroma. The tissue bridges connect the walls of the uterine cavity in all directions, which results in adhesion [2], which further result in partial or complete obstruction of the uterine cavity or the cervical canal [1]. Dilation and curettage (D&C) following miscarriage is one major cause of IUA with an incidence varies between 15 and 40% [3]. The incidence of IUA can be even as high as 40% following repeated D&C after incomplete abortion or removal of placental remnants [4]. The discrepancies in the incidence could be due to different populations and different tools used for diagnosis [5]. The presence of IUAs can lead to boosted risk of pregnancy complications including infertility, miscarriage, ectopic pregnancy, preterm delivery, abnormal placentation, intrauterine growth restriction (IUGR), preterm premature rupture of membranes (PROM) [5–7]. The pregnancy rate in patients with severe adhesion was lower than that in patients with mild adhesion [8]. IUA can be visualized by hysteroscopy, hysterosalpingography (HSG), magnetic resonance imaging (MRI), and ultrasonography including contrast sonohysterography (SHG) and 3D ultrasonography [1]. Hysteroscopy is the gold standard for diagnosis, and hysteroscopic lysis of adhesions followed with hormonal replacement therapy is the standard management of IUA [2]. However, high recurrence rate of IUA is one major obstacle for management [9]. The post-operative recurrent rate ranged from 3.1 to 23.5% among all severities of IUA and rised to 20 to 62.5% in those with severe type [5].

It is a common sense that severity of IUA is the most critical factor affecting their reproductive outcomes. Numerous evidence suggested that the conception rate, recurrence rate were boosted in women with severe IUA than in mild type [5, 8]. Therefore, an accurate evaluation of the IUA severity is one critical factor for management [10]. A number of evaluation systems have been proposed for IUA [10]. Currently, there are at least 8 different evaluation systems of IUA [10, 11]. Among them, 5 evaluation systems are commonly accepted and widely used in clinical practice in China (see supplemental Tables 1, 2, 3, 4, 5 for details). Most of the evaluation system was established through features observed during hysteroscopy. However, to date, there is no standardized, or optimal evaluation systems of IUA used to report the severity of IUA [5]. This may imply inherent deficiencies in each of the evaluation systems [10]. Moreover, none of the above evaluation systems have been validated in relation to reproductive outcomes, and few comparative analyses of different evaluation systems have been conducted [1, 2].

Therefore, to figure out an optimal evaluation system for predicting the reproductive prognosis of IUA following hysteroscopic adhesiolysis, we systematically evaluated the predictive value of live birth rate based on five different evaluation systems.

## Material and methods

### Clinical data collection

This was a retrospective cohort analysis of data from 150 patients with IUA who received transcervical resection of adhesion (TCRA) during January 2014 to December 2014 at Sun Yat -Sen Memorial Hospital. This study was approved by the local Ethics Committee (Approval No. SYSEC-KY-KS-2019-061). Informed consent and permission of acquisition of clinical data was obtained from each participant. Information including the age, history of miscarriage, the numbers of the intrauterine operations, the preoperative and postoperative endometrial thickness in the late proliferation, preoperative menstrual amount, change of menstrual amount postoperatively, the number of TCRA, hysteroscopic finding and reproductive outcomes after the surgery were collected through searching the medical record system and follow-up phone calls.

### Inclusion criteria

The inclusion criteria of IUA patients were as following, (1) women aged ≤40 years old, (2) all patients received TCRA by the same experienced surgeon, (3) all patients was diagnosed as IUA based on three-dimensional transvaginal ultrasound and hysteroscopy, (4) patients with a desire for spontaneous conception and attempted to conceive in the following 2 years after TCRA.

### Exclusion criteria

The exclusion criteria were as following, (1) breastfeeding women, (2) women with severe systemic diseases such as uncontrolled hypertension and heart disease, diabetes, liver or renal dysfunctions, immune disorders, (3) women with presence of risk factors affecting reproductive outcomes such as obesity, history of gestational diabetes, and gestational hypertension diseases, (4) women with tubal or male factors induced infertility who may require assisted reproductive techniques, (5) women with amenorrhea caused by neuroendocrine disorders, thyroid dysfunctions or endometrial tuberculosis, (6) patients with endometrial polyps, uterine submucosalmyoma or other type of uterine myoma (diameter ≥ 3 cm).

### The diagnosis and surgical treatment of IUA

The diagnosis of IUA was based on three-dimensional transvaginal ultrasound and hysteroscopy. All patients received TCRA by the same surgeon who had more than 10-year experience on hysteroscopy. All women had general anesthesia during the surgery. The surgical procedure of each woman was picture recorded in the local data center. The Olympus S70 hysteroscopy equipment was used, and either sodium chloride or mannitol was provided as perfusion medium. Adhesiolysis was performed under the direct observation under hysteroscopy and monitored under ultrasonography if necessary. The adhesion tissue was cut by needle electrodes or ring electrodes. The remaining normal endometrium tissue was protected and untouched. The surgical procedure would be finished if adhesion tissue was separated and normal uterine cavity was restored. A heart-shaped COOK intrauterine balloon stent (J-BUS-253000, COOK Medical corp., US) was inserted into and kept inside uterine cavity after TCRA until the third withdrawal bleeding. All patients were treated with sequential estrogen (Progynova 4 mg/d, Bayer Schering Pharma, France) and progesterone (Dydrogesterone, 20 mg/d, Abbott Biologicals B.V., the Netherlands) therapy for consecutive 3 cycles. The reproductive outcomes of those patients were obtained from medical record and patients were followed up through phone call interview.

### Implementation of classification systems

Here in this study, five IUA diagnosis classification criteria were compared, including The American Fertility Society classification of intrauterine adhesions (AFS, 1988) [12], European Society of Gynecological Endoscopy classification (ESGE, 1995) [13], classification system reported by March et al. (March, 1978) [14], Scoring system developed by Nasr et al. (Nasr, 2000) [15], and classification system based on concensus of Chinese experts on intrauterine adhesion diagnosis and management (Chinese, 2015) [11] (see supplemental data for details of the above classification systems). All the patients were retrospectively scored of the IUA severity according to the above five classficiation systems based on the hysteroscopic operational records and videos. The scores were graded by two trained residents by blind method. The experienced supervisor would double check the operational records if there was any discrepancy on the scoring.

### Reproductive outcomes

Women’s reproductive outcomes were checked through medical record and phone call interview. Clinical pregnancy was determined as the observation of an intrauterine pregnancy sac with live fetus through ultrasound at gestational 6–7 weeks. Live birth was determined as delivery of at least one live baby after 28-week gestation. The delivery of twin babies by one mother was defined as one live birth. Live birth rate is defined as the proportion of women with live birth out of women with a desire for spontaneous conception.

### Statistical analysis

All statistical analyses were performed using SPSS 20.0 software (IBM Corp., NY). A two-tailed probability value of *P* <  0.05 was considered as statistically significant. Normal distribution was tested for continuous variables. Descriptive parameters with a normal distribution were expressed as mean ± standard deviation (SD). Continuous variables were compared using the independent t-test. Dichotomous data were compared using χ2 test, or using Fisher’s exact test as appropriate. Ranked count data were dealt using Rank-Sum test. Correlation between dichotomous data were examined with logistic regression analysis and Spearman rank correlation analysis, and the odds ratop (OR) value and 95% confidential interval (95% CI) were calculated. Receiver Operating characteristic (ROC) curves were created for each classification system to determine their abilities to predict live birth.

## Results

### Baseline clinical characteristics of participants

**Fig. 1:**
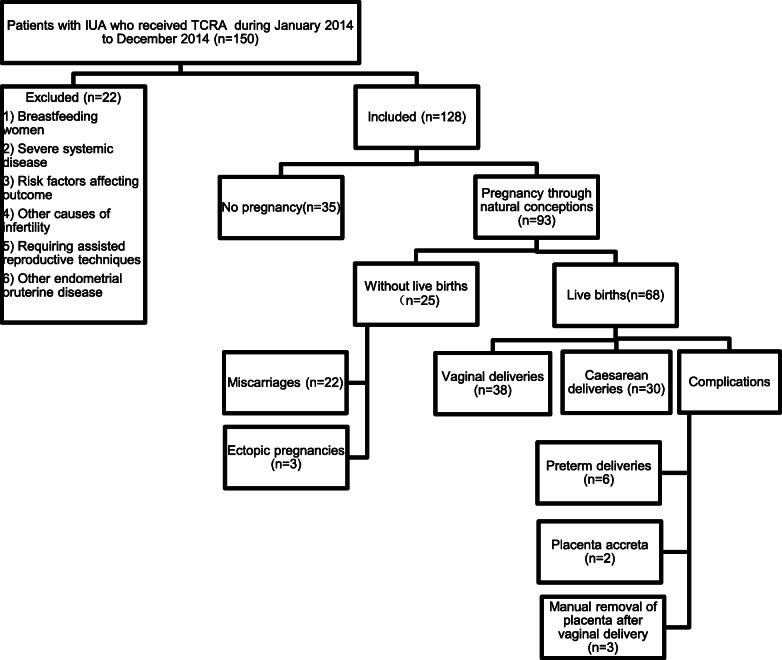
Flowchart of inclusion and follow-up of study population. Note: Women with no live birth (*n* = 60) included those with no pregnancy (*n* = 35) and those pregnant through natural conception but ended with no live birth (*n* = 25). Abbreviations: IUA, intrauterine adhesion; TCRA, transcervical resection of adhesion

As illustrated in Fig. 1, among 150 IUA patients screened, 128 patients met the inclusion criteria and attempted spontaneous conception was included in the final analysis. The inclusion and follow-up procedures of all patients were demonstrated in Fig. 1. Participants with no live birth included those with no conception (*n* = 35) and those with miscarrriage (*n* = 22) and ectopic pregnancy (*n* = 3). There were 68 women end up with live birth, and among them, 30 women had Cesarean section and the remaining 38 women had spontaneous vaginal delivery.

Baseline clinical characteristics of IUA women with and without live birth are documented in Table 1. Hypomenorrhea was the most common symptom among women with IUA (96 of 128, 75%). Women with either live birth or no live birth showed no differences regarding age, history of late miscarriage, history of recurrent spontaneous miscarriage, total number of intrauterine curettage. As noted in Table 1, more women with live birth had normal menses than those without live birth (23.5% vs. 16.7%), whereas more women without live birth had amenorrhea than those with live birth (6.7% vs. 2.9%). Majority women with live birth had no history of infertility (80.9%), and no previous TCRA (91.2%), whereas almost one in third women without live birth had history of infertility (28.3%), and at least once TCRA (31.7%). Besides, significantly more women with live birth had maximum endometrial thicknesses ≥6 mm than their counterparts (41.2% vs. 23.3%, *P* = 0.032).

**Table 1:** Comparisons of baseline clinical characteristics between women with and without live birth

	IUA with live birth (*N* = 68)	IUA without live birth (*N* = 60)	*P* value
Age (years old) (mean ± SD)	31.1 ± 5.06	32.6 ± 4.81	0.226
< 35 n(%)	55 (80.9%)	44 (73.3%)	0.398
≥ 35 n(%)	13 (19.1%)	16 (26.7%)	
Menstrual pattern before hysteroscopy n(%)			< 0.001
Hypomenorrhea	50 (73.5%)	46 (76.7%)	
Amenorrhea	2 (2.9%)	4 (6.7%)	
Normal menses	16 (23.5%)	10 (16.7%)	
History of late miscarriage n(%)			0.053
Yes	7 (10.3%)	13 (22.0%)	
No	61 (89.7%)	46 (78.0%)	
History of recurrent spontaneous miscarriage n(%)			0.648
Yes	29 (42.6%)	28 (46.7%)	
No	39 (57.4%)	32 (53.3%)	
History of Infertility n(%)			< 0.001
Primary infertility	3 (4.4%)	3 (5.0%)	
Secondary infertility	10 (14.7%)	14 (23.3%)	
No infertility	55 (80.9%)	43 (71.7%)	
Total number of uterine curettage n(%)			0.226
0	7 (10.3%)	3 (5.0%)	
1	15 (22.1%)	9 (15.0%)	
2	14 (20.6%)	19 (31.7%)	
≥ 3	32 (47.1%)	29 (48.3%)	
Number of previous TCRA n(%)			< 0.001
0	62 (91.2%)	41 (68.3%)	
1	4 (5.9%)	13 (21.7%)	
≥ 2	2 (2.9%)	6 (10.0%)	
Maximum endometrial thickness before hysteroscopy n(%)			0.032
≥ 6 mm	28 (41.2%)	14 (23.3%)	
< 6 mm	40 (58.8%)	46 (76.7%)	

Abbreviations: *IUA* intrauterine adhesion, *TCRA* transcervical resection of adhesion

Table 2 compared the observations under hysteroscopy between women with live birth and no live birth. Even though the post-operative menstruation pattern showed no differences between the two groups, the volume of uterine cavity involved, visualization of uterine horns, visualization of tubal ostia, type of adhesion were notably differed between live birth and no live birth groups (*P* <  0.001, see Table 2 for details). Hence, the extent of adhesion might contribute notably to the live birth chance.

**Table 2:** Comparisons of observations under hysteroscopy between women with and without live birth

	IUA with live birth (*N* = 68)	IUA without live birth (*N* = 60)	*P* value
Volume of uterine cavity involved n(%)			0.001
≤ 1/4	24 (35.3%)	6 (10.0%)	
1/4–1/3	19 (27.9%)	21 (35.0%)	
1/3–2/3	14 (20.6%)	12 (20.0%)	
2/3–3/4	6 (8.8%)	12 (20.0%)	
> 3/4	5 (7.4%)	9 (15.0%)	
Visualization of Uterine horns n(%)			< 0.001
Both visualized	33 (48.5%)	13 (21.7%)	
One side visualized	20 (29.4%)	21 (35.0%)	
Neither visualized	15 (22.1%)	26 (43.3%)	
Visualization of tubal ostia n(%)			< 0.001
Both visualized	24 (35.3%)	14 (23.3%)	
One side visualized	29 (42.6%)	22 (36.7%)	
Neither visualized	15 (22.1%)	24 (40.0%)	
Type of adhesion n(%)			< 0.001
Membranous adhesion	9 (13.2%)	10 (16.7%)	
Fibrous adhesion	42 (61.8%)	26 (43.3%)	
Myogenic adhesion	17 (25.0%)	24 (40.0%)	
Postoperative menstruation pattern n(%)			0.146
Increased amount	66 (97.1%)	54 (90.0%)	
No increased amount	2 (2.9%)	6 (10.0%)	

Abbreviations: *IUA* intrauterine adhesion

### Distribution of live birth rate of patients with different severities of IUA

In the present study, the severities of 128 cases with IUA were evaluated using AFS, ESGE, March, Nasr, and Chinese classification system, respectively. As clearly demonstrated in Table 3, the live birth rates reduced significantly as the severity of IUA increased regardless of which classification system was used to evaluate the severity of IUA.

**Table 3:** Live birth rates of different severities of IUA defined by different classification systems

Evaluation systems	Grades	N (*n* = 128)	IUA with live birth (*n* = 68)	IUA without live birth (*n* = 60)	*P* value
AFS classification	I	26	18 (69.2%)	8 (30.8%)	< 0.001
	II	72	43 (59.7%)	29 (40.3%)	
	III	30	7 (23.3%)	23 (76.7%)	
ESGE classification	Mild	12	10 (83.3%)	2 (16.7%)	< 0.001
	Moderate	78	46 (59.0%)	32 (41.0%)	
	Severe	38	12 (31.6%)	26 (68.4%)	
March classification	Mild	16	12 (75.0%)	4 (25.0%)	< 0.001
	Moderate	64	39 (60.9%)	25 (39.1%)	
	Severe	48	17 (35.4%)	31 (64.6%)	
Nasr classification	Mild	11	9 (81.8%)	2 (18.2%)	< 0.001
	Moderate	53	37 (69.8%)	16 (30.2%)	
	Severe	64	22 (34.4%)	42 (65.6%)	
Chinese classification	Mild	15	11 (73.3%)	4 (26.7%)	< 0.001
	Moderate	87	52 (59.8%)	35 (40.2%)	
	Severe	26	5 (19.2%)	21 (80.8%)	

Abbreviations: *IUA* intrauterine adhesion, *AFS* The American Fertility Society, *ESGE* European Society of Gynecological Endoscopy

### Correlation of different evaluation systems and the live birth rates of postoperative IUA

The correlations between these five evaluation systems and live birth rates were determined by Spearman rank correlation analysis. The correlation coefficients of AFS, ESGE, March, Nasr, Chinese classification and the live birth rates were 0.313, 0.313, 0.288, 0.380, and 0.336, respectively (Table 4, *P* <  0.001). Moreover, the correlation between live birth rate the all five evaluation systems were adjusted for menstrual pattern before hysteroscopy and history of infertility. As demonstrated in Table 4, among women with hypomenorrhea and amenorrhea, as well as women with no infertility, the severities determined by all five evaluation systems were correlated with live birth rate (*P* <  0.001). such correlations were not observed among with normal menses and those with infertility history. Although each classification system was related with the live birth rate, the correlation coefficients was small to medium, indicating that other factors may also contribute to the live birth outcome. Among them, Nasr classification showed the highest correlation coefficient (*r* = 0.380, *P* <  0.001).

**Table 4:** Correlation analyses between different evaluation systems and live birth rate

Classification systems	Correlation coefficient	*P* value
All cases		
AFS classification	0.313	< 0.001
ESGE classification	0.313	< 0.001
March classification	0.288	< 0.001
Nasr classification	0.380	< 0.001
Chinese classification	0.336	< 0.001
Menstrual pattern before hysteroscopy		
Hypomenorrhea & amenorrhea		
AFS classification	0.438	< 0.001
ESGE classification	0.335	< 0.001
March classification	0.321	< 0.001
Nasr classification	0.460	< 0.001
Chinese classification	0.429	< 0.001
Normal menses		
AFS classification	−0.132	0.522
ESGE classification	0.273	0.178
March classification	0.134	0.514
Nasr classification	0.231	0.255
Chinese classification	0.063	0.761
History of Infertility n(%)		
Infertility		
AFS classification	0.064	0.739
ESGE classification	0.030	0.873
March classification	0.172	0.364
Nasr classification	0.000	1.000
Chinese classification	0.283	0.129
No infertility		
AFS classification	0.417	< 0.001
ESGE classification	0.399	< 0.001
March classification	0.343	< 0.001
Nasr classification	0.489	< 0.001
Chinese classification	0.319	< 0.001

Abbreviations: *IUA* intrauterine adhesion, *AFS* The American Fertility Society, *ESGE* European Society of Gynecological Endoscopy

### Univariate and multivariate logistic regression analyses of different evaluation systems on the live birth rate of postoperative IUA

The relationship between live birth rate and these five evaluation systems were further examined with univariate logistic regression analyses respectively. Data showed in Table 5 also confirmed that the live birth rates were closely associated with the severities of IUA determined using each evaluation system (Table 5). Considering the impact of other compounding factors, including age, menstrual patter before hysteroscopy, history of infertility, number of previous TCRA, and endometrial thickness before hysteroscopy, multivariate logistic regression analyses were also performed to confirm the independent influences of different evaluation systems on the live birth rate. As indicated in Table 5, each classification system showed independent influence on the live birth outcomes, and among them, Nasr classification demonstrated the highest OR (OR, 6.523; 95% CI, 2.612, 18.263).

**Table 5:** Univariate and multiple logistic regression analysis of different evaluation systems on live birth rates of postoperative IUA

	Univariate analysis			Multiple Regression analysis		
	OR	95% CI	*P* value	OR	95% CI	*P* value
AFS classification	4.043	1.507, 10.837	0.006	3.062	1.236, 7.604	0.032
ESGE classification	4.563	1.701, 12.244	0.003	3.263	1.305, 8.672	0.016
March classification	3.209	1.267, 8.125	0.014	3.101	2.115, 6.441	0.019
Nasr classification	8.281	2.732, 25.103	0.000	6.523	2.612, 18.263	0.000
Chinese classification	7.629	2.314, 25.179	0.001	5.472	2.021, 17.618	0.001

Abbreviations: *IUA* intrauterine adhesion, *AFS* The American Fertility Society, *ESGE* European Society of Gynecological Endoscopy, *OR* odds ratio, *95% CI* 95% confidential index

### The ROC curve of different evaluation systems to predict the live birth rate of IUA

**Fig. 2:**
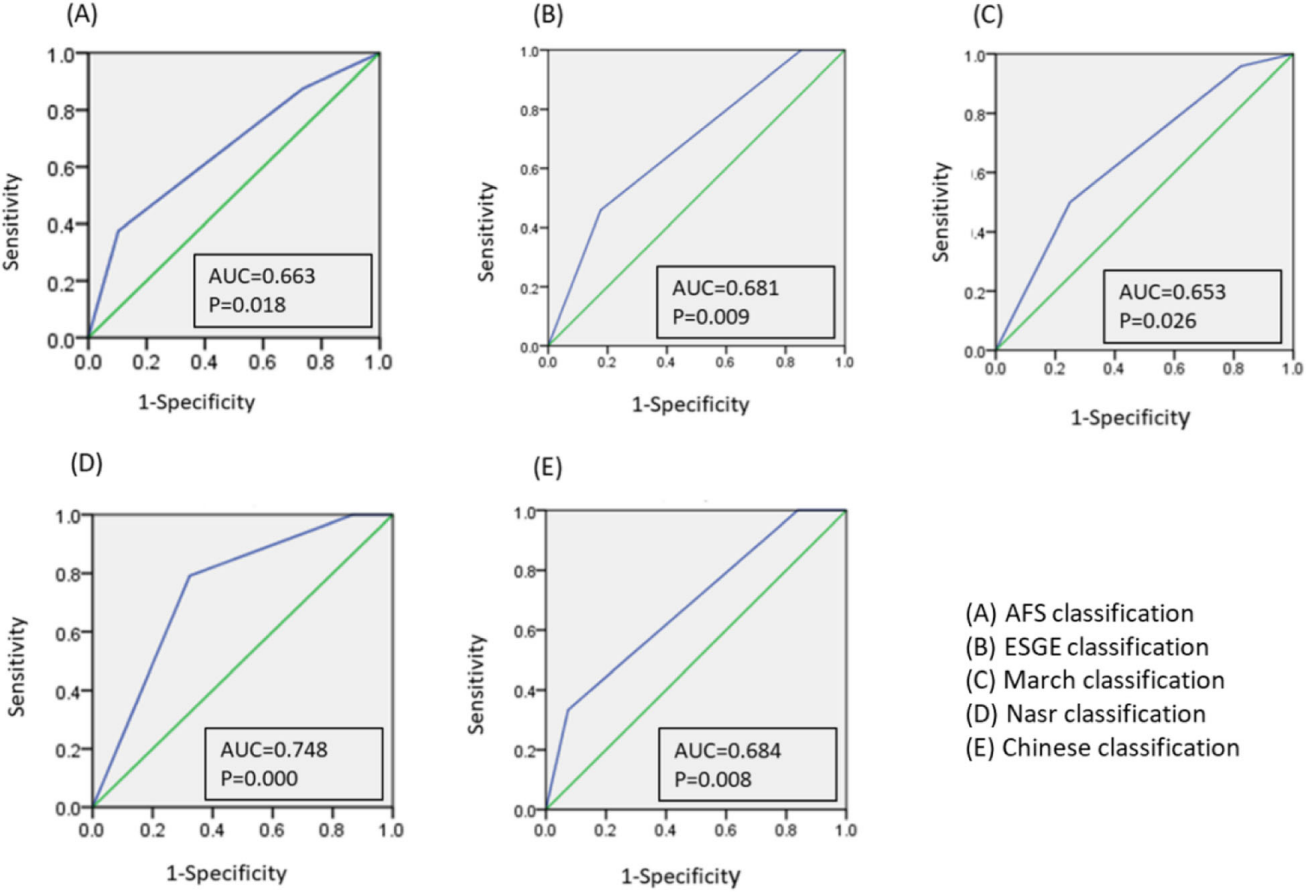
ROC curves of different evaluation systems to predict the live birth rates of IUA women. Abbreviations: ROC, receiver operating characteristic curve; IUA, intrauterine adhesion

The predictive value of live birth rate using each evaluation system was determined with ROC curve (Fig. 2). The AUCs of AFS, ESGE, March, Nasr and Chinese classification systems were 0.663, 0.681, 0.653, 0.748 and 0.684, respectively (Fig. 2). It is of interest to note that the Nasr classification, again, with the highest AUC (0.748), had the highest predicting value. Table 6 provided detailed sensitivities, specificities, positive and negative predictive values of each classification system. Among them, Nasr classification system showed highest sensitivity (81.8%) and negative predictive value (96.7%) when divide the system into mild IUA vs. moderate and severe IUA; and Chinese classification system showed highest specificity (80.8%) and positive predictive value (92.6%) when divide the system into mile and moderate IUA vs. severe IUA.

**Table 6:** Sensitivity, specificity, positive predictive value and negative predictive value of each classification system of IUA

	Classification	Sensitivity	Specificity	Positive predictive value	Negative predictive value
AFS	Stage I vs. Stage II + III	69.2%	51.0%	26.5%	86.7%
	Stage I+ II vs. Stage III	62.2%	76.7%	89.7%	38.3%
ESGE	Mild vs. Moderate +Severe	83.3%	50.0%	14.7%	96.7%
	Mild + Moderate vs. Severe	62.2%	68.4%	82.4%	43.3%
March	Mild vs. Moderate +Severe	75.0%	50.0%	17.6%	93.3%
	Mild + Moderate vs. Severe	63.8%	64.6%	75.0%	51.7%
Nasr	Mild vs. Moderate +Severe	81.8%	49.6%	13.2%	96.7%
	Mild + Moderate vs. Severe	71.9%	65.6%	67.6%	70.0%
Chinese	Mild vs. Moderate +Severe	73.3%	49.6%	16.2%	93.3%
	Mild + Moderate vs. Severe	61.8%	80.8%	92.6%	35.0%

Abbreviations: *IUA* intrauterine adhesion, *AFS* The American Fertility Society, *ESGE* European Society of Gynecological Endoscopy

## Discussion

In the present study, the reproductive outcomes of 128 cases of IUA with different severities were reported. The predictive values of live birth rate using five evaluation systems including AFS, ESGE, March, Nasr and Chinese classification systems were compared to figure out an optimal evaluation system for IUA. As expected, the live birth rate following spontaneous conception closely related with the severities of IUA. Furthermore, although all five classification systems were capable to predict live birth rate, Nasr classification, with the highest AUC, might be the optimal prediction system for live birth after natural conception.

Previous reports implied that the severity of adhesions was negatively correlated with the reproductive outcome after hysteroscopic adhesiolysis [16, 17]. There were 83.3% of women with mild adhesions achieved live births, whereas only 25% women with severe adhesion had live births [16]. American Association of Gynecological Laparoscopists reported a practice guideline on intrauterine adhesions which was developed in collaboration with ESGE in 2017. This guidelines also proposed that IUA should be accurately classified because prognosis of live birth is highly related to severities of adhesion, and it is impossible to endorse any specific system because it is difficult to compare various evaluation systems between studies [10]. Therefore, an accurate assessment of the IUA severities is one key factor for management.

The five evaluation systems in the present study evaluate IUA severities mainly based on hysteroscopic findings, but the specific evaluation items involved in the classifications are different. The earliest evaluation system of IUA, March classification [14] classifies adhesions as mild, moderate or severe types only based on the extent and type of adhesions observed during hysteroscopy, which is simple and practical. AFS classification [12] defined the severities of IUA into three stages (I - III) based on a combined evaluation of hysteroscopic or HSG findings and the menstrual pattern. It was the first evaluation system which takes the menstrual pattern into consideration. ESGE classification [13], classifies IUA as stages I to V with subtypes (seven stages in all) based on hysteroscopy and HSG findings. This classification is more complex but more accurate in the aspect of the extent, location and type of adhesions, hence experienced operational skill of hysteroscopy operator is required. Nasr classification [15] is the first classification that incorporate hysteroscopic findings with menstrual pattern as well as obstetric history, and correlates them with the prognosis of IUA. Nasr et al. considered the menstrual pattern as an important prognostic point. Besides, they believed that the type of adhesions, especially the tubular cavity, was more decisive to the severity and prognosis of IUA. The latest Chinese classification [11] was proposed based on AFS and ESGE classifications, including menstrual pattern and reproductive history, besides of observations under hysteroscopy.

It is reasonable to include risk factors affecting the reproductive outcomes as the basis of IUA classcification systems. The extent of uterine cavity involved and type of adhesions are included in all five classifications. This may be due to the fact that the extent and location of IUA are the dominant factors affecting reproductive outcomes [18], as also suggested in our results (Table 2). Taylor et al. [19] suggested that filmy IUA may lead to infertility, in addition to dense IUA. Nasr et al. believed that the prognosis was associated with the type of adhesions and whether or not they cover the tubal ostia [15]. An early study reported that a classification of IUA based on both adhesion types and of uterine cavity occlusion extent, is valuable in the prediction of reproductive outcome [20]. It is not clear the live birth outcomes of each IUA adhesion type. Here, in the present study, we did find that women with fibrous adhesion end up with high live birth rate.

Yu et al. [21] and Roy et al. [22] suggested that the menstrual pattern after hysteroscopic surgery had a significant impact on reproductive prognosis but no such correlation were observed between the menstrual pattern before hysteroscopic surgery and the prognosis of IUA. This could be explained by the fact that the menstrual pattern after surgery reflects the remaining amount of endometrium available for regeneration, which is critical for the implantation ability of endometrium [15, 21]. However, conflicting with an earlier report [23], March et al. concluded that there was no perfect correlation between the extent of IUA and menstrual pattern [24], hence menstrual pattern was not contained in its classification system. Still, AFS, Nasr and Chinese evaluation systems included the menstrual pattern before hysteroscopic surgery as one factor to determine the IUA classification. Here in this study, among the 128 women who desired to conceive spontaneously in the following 24 months after TCRA, 93.8% of them achieved improvement in menstruation pattern, which is similar to He’s report that 91.30% of IUA patients showed with improvement in menstrual amount after TCRA [25]. However, similar proportions of women with and without live birth showed increased amount of menstrual blood following TCRA, which might indicate that improvement in menstrual amount is not closely related with reproductive outcomes, and only menstrual pattern before hysteroscopy is a prognostic factor of live birth outcome. The association between menstrual pattern and reproductive outcome of IUA is still controversial, which might be because the report of menstrual pattern is quite subjective based on patients’ own evaluation.

The maximum endometrial thickness is another critical factor predicting the live birth outcomes of IUA patients. IUA occurs due to the disruption of the basalis layer of the endometrium [2]. Malhotra et al. believed that thin and damaged endometrium lost its endometrial receptivity and hampers implantation in women with Asherman’s syndrome [26]. In their series, by analyzing the endometrial thickness and Doppler flows in infertile women with IUA both pre- and post-hysteroscopic, they observed patients with flimsy adhesion achieved an improvement in the endometrial thickness, while patients with dense adhesions had no change in the endometrial thickness and blood flow and the pregnancy outcome remained poor. Bhandari et al. [27] checked changes in endometrial thickness with ultrasonography and found that the pregnancy outcomes of IUA patients were associated with postoperative endometrial echo pattern, which reflected that endometrial function after hysteroscopic adhesiolysis plays an important role in determining the reproductive outcome. Baradwan et al. reported that pregnancy rates were higher in endometrial thickness > 5 mm group (80%) than group with < 5 mm endometrium (38.46%) [28]. Researchers [29, 30] also found that endometrial thickness was a significant and independent predictor of live birth after the in vitro fertilization/intracytoplasmic sperm injection (IVF/ICSI) treatment, and the live birth rate rise with endometrial thickness increasing. Live birth rate was significantly reduced when endometrial thickness less than 8 mm, and no adverse pregnancy outcome was observed when endometrial thickness more than 14 mm [31]. However, lower implantation and pregnancy rates when endometrial thickness more than 14 mm on the day of HCG administration [32]. Controversially, some researchers concluded that endometrial thickness has limited capacity to identify women who have a low pregnancy rate after IVF [33], and pregnancy outcome could not be predicted by endometrial thickness alone [34]. Overall, adequate endometrial thickness is required for successful pregnancy. According to the analysis above, we believe that endometrial thickness before and after adhesiolysis can be taken into consideration as prognosis points for live birth. Nevertheless, among the five evaluation systems in the present study, only Chinese classification includes the endometrial thickness as one of the parameters. Further studies are required to determine the optimal endometrial thickness for satisfactory reproductive outcomes of IUA women.

An ideal and clinically practical evaluation system of IUA should be simple, reproducible, quantifiable, and capable of predicting the reproductive prognosis. To date, no evaluation system obtained universal recognition, which might reflect inherent deficiencies in all of these proposed systems. In the present study, all the five evaluation systems were demonstrated to be valuable of predicting live birth. Among them, Nasr classification system demonstrated the highest AUC value, which might be due to its inclusion of most associated factors with live birth. On the contrary, March classification, which was established four decades ago, showed a low predictive value of live birth, which could be attributed to the simplified evaluation approach based only on hysteroscopic features.

It is difficult to compare live birth rates with other studies due to the different durations of follow-up, variable proportions of different severities, different post-surgery treatments and retrospective bias. One study reported that the live birth rate after hysteroscopic treatment was 63.7% (79/124) with 14-year follow-up [35], whereas the live birth rate in our series was 53.1% (68 /128), which may be in part due to the shorter duration of follow-up. Another study from one hospital in China, with the similar inclusion criteria and management of IUA to our study, reported a similar live birth rate of 42.2% (140/332) as in our study after following up for 27 ± 9 months [36]. A pooled live birth rate, 46.3% (696/1504), was reported from a systematic review containing 36 studies, which was also similar to our study [37].

To our knowledge, this is the first study to compare the predictive value of live birth rate based on the five evaluation systems including AFS, ESGE, March, Nasr and Chinese classifications. However, this study is limited by its retrospective design and relatively small number of patients included. Besides, certain clinical characteristics, including the gravidity, parity, and body mass index was not obtained due to its retrospective nature. Further, we should also acknowledge the the evaluation for women with multiple TCRA may not represent the original severity of IUA, and may bring potential bias on the interpretation of the outcomes. And finnally, the AUC of most classification systems was not satisfactory enough (AUC <  0.7), indicating other risk factors may also contributing to the live birth outcomes.

## Conclusion

AFS, ESGE, March, Nasr and Chinese classifications of IUA were demonstrated to be capable of predicting live birth following TCRA, although the predictive capacities might be limited. Among them, Nasr classification might be the optimal evaluation system for predicting live birth following TCRA through natural conception.

## Supplementary Information

**Additional file 1: Table S1.** The American Fertility Society (AFS) classification of intrauterine adhesions, 1988. **Table S2.** Classification by March 1978. **Table S3.** European Society of Gynecological Endoscopy (ESGE) classification of IUAs (1995 version). **Table S4.** Scoring by Nasr 2000. **Table S5.** Chinese intrauterine adhesions diagnosis classification criteria in 2015.

## Data Availability

The datasets used and/or analysed during the current study are available from the corresponding author on reasonable request.
