# Supplementary material for: Predictive value of live birth rate based on different intrauterine adhesion evaluation systems following TCRA
Source: Reprod Biol Endocrinol. 2021 Jan 22;19:13. doi: 10.1186/s12958-021-00697-1 (PMC7821669; doi:10.1186/s12958-021-00697-1)
Supplement: Supplementary file 1 — Additional file 1: Table S1. The American Fertility Society (AFS) classification of intrauterine adhesions, 1988. Table S2. Classification by March 1978. Table S3. European Society of Gynecological Endoscopy (ESGE) classification of IUAs (1995 version). Table S4. Scoring by Nasr 2000. Table S5. Chinese intrauterine adhesions diagnosis classification criteria in 2015. [file 12958_2021_697_MOESM1_ESM.docx]

**Supplement**

Supplemental Table 1 The American Fertility Society (AFS) classification of intrauterine adhesions, 1988.

| **Classification** | | **Condition** | | | |
| --- | --- | --- | --- | --- | --- |
| Extent of Cavity Involved | | < l/3 | | 1/3–2/3 | > 2/3 |
|  |  | 1 | | 2 | 4 |
| Type of Adhesions | | Filmy | | Filmy & Dense | Dense |
|  |  | 1 | | 2 | 4 |
| Menstrual Pattern | | Normal | | Hypomenorrhea | Amenorrhea |
|  |  | 0 | | 2 | 4 |
| **Prognostic classification** | | | | **HSG^a^ score** | **Hysteroscopy score** |
| Stage l | (Mild) | | 1-4 | __________ | __________ |
| Stage ll | (Moderate) | | 5-8 | __________ | __________ |
| Stage lll | (Severe) | | 9-12 | __________ | __________ |

^a^ All adhesions should be considered dense.

Supplemental Table 2 Classification by March 1978.

| **Classification** | **Involvement** |
| --- | --- |
| Mild | < 1/4 of uterine cavity involved;  Thin or filmy adhesions;  Ostial areas and upper fundus minimally involved or clear. |
| Moderate | 1/4 to 3/4 of uterine cavity involved;  No agglutination of uterine walls-adhesions only;  Ostial areas and upper fundus partially occluded. |
| Severe | > 3/4 of uterine cavity involved;  Agglutination of walls or thick bands;  Ostial areas and upper cavity occluded. |

Supplemental Table 3 European Society of Gynecological Endoscopy (ESGE) classification of IUAs (1995 version).

| **Grade** | **Extent of intrauterine adhesions^a^** |
| --- | --- |
| I | Thin or filmy adhesions  Cornual areas normal  Easily ruptured by hysteroscope sheath alone |
| II | Singular dense adhesion  Connecting separate areas of the uterine cavity  Visualization of both tubal ostia possible  Cannot be ruptured by hysteroscope sheath alone |
| IIa | Occluding adhesions only in the region of the internal cervical os^b^  Upper uterine cavity normal |
| III | Multiple dense adhesions  Connecting separate areas of the uterine cavity  Unilateral obliteration of ostial areas of the tubes |
| IV | Extensive dense adhesions with (partial) occlusion of the uterine cavity  Both tubal ostial areas (partially) occluded |
| Va | Extensive endometrial scarring and fibrosis in combination with grade I or grade II adhesions |
| Vb | Extensive endometrial scarring and fibrosis in combination with grade III or grade IV adhesions^b^ |

^a^ From findings at hysteroscopy and hysterography.

^b^ Only to be classified during hysteroscopic treatment.

ESGE classification is modified in our study: grade I was considerd to be mile adhesions, grade II -III was considerd to be moderate adhesions, and grade IV -V represented severe adhesions.

Supplemental Table 4 Scoring by Nasr 2000.

| **Hysteroscopic findings** |  | **Score** |
| --- | --- | --- |
| Isthmic fibrosis |  | 2 |
| Filmy adhesions | Few | 1 |
|  | Excessive(i.e., >50%, of the cavity | 2 |
| Dense adhesions | Single band | 2 |
|  | Multiple bands (i.e., >50% of the cavity) | 4 |
| Tubal ostium | Both visualized | 0 |
|  | Only one visualized | 2 |
|  | Both not visualized | 4 |
| Tubular cavity (sound less than 6cm) | | 10 |
| Menstrual pattern | Normal | 0 |
|  | Hypomenorrhea | 4 |
|  | Amenorrhea | 8 |
| Reproductive performance | Good obstetric history | 0 |
|  | Recurrent pregnancy loss | 2 |
|  | Infertility | 4 |
| 0–4 = Mild (good prognosis); 5–10 = moderate (fair prognosis); 11–22 = severe (poor prognosis). | | |

Supplemental Table 5 Chinese intrauterine adhesions diagnosis classification criteria in 2015.

| **Evaluation index** | **Condition** | **Score** |
| --- | --- | --- |
| Extent of Cavity Involved | <l/3 | 1 |
|  | 1/3–2/3 | 2 |
|  | >2/3 | 4 |
| Type of adhesion | Membranous adhesion | 1 |
|  | Fibrous adhesion | 2 |
|  | Myogenic adhesion | 4 |
| Tubal ostium | Only one visualized | 1 |
|  | Both not visualized | 2 |
|  | Barrel uterine cavity; the corneal area of uterine both not visualized | 4 |
| Thickness of endometrium | ≥7mm | 1 |
|  | 4-6mm | 2 |
|  | ≤3mm | 4 |
| Menstrual pattern | Hypomenorrhea(more than 1/2 reduction) | 1 |
|  | Hypomenorrhea(drip bleeding) | 2 |
|  | Amenorrhea | 4 |
| History of adverse pregnancy | Have a spontaneous abortion | 1 |
|  | Recurrent abortion | 2 |
|  | Infertility | 4 |
| History of uterine curettage | Induced abortion | 1 |
|  | Curettage in early pregnancy | 2 |
|  | Curettage in mid to late pregnancy | 4 |
| 0-8= Mild; 9-18= moderate; 19-28 = severe | | |
